# Supplementary material for: What factors determine users’ knowledge payment decisions? A mixed-method study
Source: PLoS One. 2023 Jul 7;18(7):e0287560. doi: 10.1371/journal.pone.0287560 (PMC10328235; doi:10.1371/journal.pone.0287560)
Supplement: Attachment — Submitted filename: PONE-D-22-27874(Rebuttal Letter)-Original.docx [file pone.0287560.s002.docx]

**Rebuttal Letter**

Dear Editor and Reviewers,

Thank you for allowing us to revise the manuscript and address the reviewers’ comments.

Please see the following response letter, including our point-by-point responses to the editor and reviewers’ comments.

We hope that you are satisfied with this letter and that the manuscript will now be suitable for publication.

Sincerely,

On behalf of all authors,

Prof. Dr. Sang-Joon Lee

**From the academic editor:**

1. **Please ensure that your manuscript meets PLOS ONE's style requirements, including those for file naming.**

Response: PLOS One Template is used. File naming is changed to PONE-D-22-27874.

2. **You indicated that ethical approval was not necessary for your study. We understand that the framework for ethical oversight requirements for studies of this type may differ depending on the setting and we would appreciate some further clarification regarding your research. Could you please provide further details on why your study is exempt from the need for approval and confirmation from your institutional review board or research ethics committee (e.g., in the form of a letter or email correspondence) that ethics review was not necessary for this study? Please include a copy of the correspondence as an ""Other"" file.**

Response: Institutional Review Board approval is required. However, we indicated that ethical approval is not necessary for our study because we strongly believe that ethical review is only needed if researchers are unable to obtain consent, if the case report contains sensitive information, or if there is a potential risk to patient privacy. We do not consider such sensitive information in our research. We strongly believe that the use of existing data collections or records that contain only non-identifiable data, e.g., survey response data, publicly accessible records, archives, or publications, are exempted from ethical approval. Anyway, we have already got ethical approval from our institution that we include for your kind consideration. The number is as follow:

1040198-230207-HR-010-02

3. **We note that the grant information you provided in the ‘Funding Information’ and ‘Financial Disclosure’ sections do not match. When you resubmit, please ensure that you provide the correct grant numbers for the awards you received for your study in the ‘Funding Information’ section.**

Response: Thank you for your comments. We have already removed any funding-related text from the manuscript. Our funding information is:

This research was supported by the MSIT (Ministry of Science and ICT), Korea, under the Innovative Human Resource Development for Local Intellectualization support program (IITP-2022-RS-2022-00156287) supervised by the IITP (Institute for Information & Communications Technology Planning & Evaluation). This work was supported by an IITP grant funded by the Korean government (MSIT) (No.2022-0-01203, Regional strategic Industry convergence security core talent training business).

4. **Thank you for stating the following in the Acknowledgments Section of your manuscript:**

**“This research was made possible by a funding from the Korean government (MSIT)'s-(No. 2019-0-01343)- Institute of Information and Communications Technology Planning and Evaluation (IITP).”**

**We note that you have provided funding information that is not currently declared in your Funding Statement. However, funding information should not appear in the Acknowledgments section or other areas of your manuscript. We will only publish funding information present in the Funding Statement section of the online submission form.**

Response: We have already removed the funding information from the acknowledgments section. Though our funding information has already been moved from this section as a financial disclosure, this is why we also omitted the acknowledgment from our manuscript. However, we would like to express our gratitude to those supporting us during this research. For your consideration, our updated acknowledgments are given below (this can also be found on lines 867-873 of page 22).

We would like to express our gratitude to MSIT (Ministry of Science and ICT), Korea, under the Innovative Human Resource Development for Local Intellectualization support program (IITP-2022-RS-2022-00156287), supervised by the IITP (Institute for Information & Communications Technology Planning & Evaluation) for the unconditional support of this research. We are also immensely grateful to the IITP grant, funded by the Korean government (MSIT) (No.2022-0-01203, Regional strategic Industry convergence security core talent training business), for supporting us during this research.

5. **Thank you for stating the following financial disclosure:
   “This research was funded by supported by the BK21 FOUR Program, funded by the Ministry of Education (MOE, Korea) and the National Research Foundation of Korea (NRF).”
   Please state what role the funders took in the study.  If the funders had no role, please state: ""The funders had no role in study design, data collection and analysis, decision to publish, or preparation of the manuscript.""
   If this statement is not correct you must amend it as needed.
   Please include this amended Role of Funder statement in your cover letter; we will change the online submission form on your behalf.**

Response: We have amended this as follows:

This research was supported by the MSIT (Ministry of Science and ICT), Korea, under the Innovative Human Resource Development for Local Intellectualization support program (IITP-2022-RS-2022-00156287) supervised by the IITP (Institute for Information & Communications Technology Planning & Evaluation). This work was supported by an IITP grant funded by the Korean government (MSIT) (No.2022-0-01203, Regional strategic Industry convergence security core talent training business). The funders had no role in the study design, data collection and analysis, publication decision, or manuscript preparation.

6. **In your Data Availability statement, you have not specified where the minimal data set underlying the results described in your manuscript can be found. PLOS defines a study's minimal data set as the underlying data used to reach the conclusions drawn in the manuscript and any additional data required to replicate the reported study findings in their entirety. All PLOS journals require that the minimal data set be made fully available**.

Response: Yes - all data are fully available without restriction. Please see the following link to the data on Google Drive:

<https://drive.google.com/file/d/1YDpPGnlg0J3HCPePVL5LTt3-6Lwy4KqQ/view?usp=sharing>

7. **PLOS requires an ORCID iD for the corresponding author in Editorial Manager on papers submitted after December 6th, 2016. Please ensure that you have an ORCID iD and that it is validated in Editorial Manager. To do this, go to ‘Update my Information’ (in the upper left-hand corner of the main menu), and click on the Fetch/Validate link next to the ORCID field. This will take you to the ORCID site and allow you to create a new iD or authenticate a pre-existing iD in Editorial Manager.**

**Please review your reference list to ensure that it is complete and correct. If you have cited papers that have been retracted, please include the rationale for doing so in the manuscript text, or remove these references and replace them with relevant current references. Any changes to the reference list should be mentioned in the rebuttal letter that accompanies your revised manuscript. If you need to cite a retracted article, indicate the article’s retracted status in the References list and also include a citation and full reference for the retraction notice.**

Response: Thank you very much for your suggestion. We have already updated our ORCID ID for the corresponding author in the Editorial Manager. We have also updated our reference list based on the journal requirements.

**Reviewers’ Comments:**

We have modified the manuscript to address the points made by the reviewers. We agree with the comments on all accounts and believe that the manuscript is now more readable and informative, and its conclusions are more useful to the public.

1. **Is the manuscript technically sound, and do the data support the conclusions?**

**Reviewer #1: Yes
Reviewer #2: Yes**

Response: Thank you. We also strongly believe that our manuscript is technically sound, and the data of this paper is supported the conclusions.

2. **Has the statistical analysis been performed appropriately and rigorously?
   Reviewer #1: Yes
   Reviewer #2: Yes**

Response: Thank you. We tried our best to perform the statistical analysis appropriately and rigorously.

3. **Have the authors made all data underlying the findings in their manuscript fully available?**

**Reviewer #1: Yes
Reviewer #2: No**

Response: Thank you for pointing this out. Yes - all data are now fully available without restriction. Please see the following link to the data on Google Drive:

<https://drive.google.com/file/d/1YDpPGnlg0J3HCPePVL5LTt3-6Lwy4KqQ/view?usp=sharing>

4. **Is the manuscript presented in an intelligible fashion and written in standard English?**

**Reviewer #1: No
Reviewer #2: No**

Response: Thank you for this suggestion. An English editing service has revised the article. The corrections can be found in the revised manuscript using the track changes function.

5. **Review Comments to the Author**

**Reviewer #1:**

- **The article would benefit from a further revision by an English speaker and a copyeditor. Moreover, there are some typing errors, such as the absence of spaces between words throughout the entire manuscript.**

Response: Thank you for this suggestion and for pointing this out. An English editing service has revised the article, and the corrections can be found in the revised manuscript using the track changes function.

- **About the study design, the authors declare that they have conducted a mixed-method study, also specifying it in the title. However, the results of the qualitative study are not even hinted at. Based on the title of the paper, one would expect to read both qualitative and quantitative results. For this reason, I suggest changing the title of the paper or at least synthesizing what emerged from the qualitative study. In fact, all the variables that the authors have chosen to include in the model seem strongly anchored to the literature on the subject; therefore, one would wonder what contribution the qualitative study gave to the elaboration of the tested model.**

Response: Thank you for this suggestion. It would have been interesting to explore this aspect. However, changing our title is impossible as we have conducted a mixed-method study. The methodology section includes our explanation regarding the qualitative method (highlighted in orange). For your kind consideration, we have copied and pasted this section below (it can also be found on lines 393-538 on pages 10-14):

In this study, qualitative research was used to study the same problem using different methods so that the researchers could improve their self-confidence in the accuracy of the research results. Using qualitative research methods, a complete description of the research questions is provided to optimize the results.

Interviews are one of the most common qualitative data collection approaches, enabling researchers to gain deep insights from rich narratives [36]. Following [37], to promote data reliability in a qualitative study, the interviewer asked each question in a prescribed order. Meanwhile, to ensure inferential validity, including interpretive validity and confirmability, we attempted to accurately understand interviewees’ thoughts, views, feelings, and intentions by analyzing their discussions as recorded during the interview. We also confirmed and corroborated the interview results by cross-checking them with two colleagues to ensure their confirmability.

We used FGI to gather data on ZhihuLive users in China. We displayed posters and spread social recommendations to attract users to participate in our discussion. The focus group consisted of six users, which helped encourage a meaningful conversation [38]. The respondents consisted of four male and two female users who had 3 to 18 months of service experience, indicating that they are very familiar with the various situations in which the service is used. Their ages ranged from 20 to 30 years, representing the majority of service users. Also, the informants included university students, government and company employees, and an individual business owner.

We carefully designed the interview following [39]. The interview was carried out by a marketing lecturer and was conducted in a relaxed atmosphere in which participants were encouraged to talk openly.

The interview was divided into three sections. In the first part, we invited participants to share their service use experiences. The purpose of this initial stage was to arouse informants’ enthusiasm through a light-hearted question and determine how they thought and felt about the ZhihuLive service. In the second section, we discussed the particulars of the ZhihuLive platform and their perspectives on service through the lens of individual psychology. In the third section, we discussed the factors that influence the use of knowledge payment services, their frequency of use, and recommendations for any business model innovations. The insights from this section contribute to the subsequent development of hypotheses based on theoretical rationales from prior research.

The interview lasted nearly one hour and was smartphone-recorded and transcribed verbatim. Following the principles of [40], the content analyses of the recording and transcript were performed separately by two colleagues unaware of the specific research framework and hypotheses to avoid potential bias.

According to the interviews and research, ZhihuLive is characterized by applications in a short time, effective utilization of this time, and an ability to provide valuable information to users to solve their problems and assist them in learning new knowledge. The users found the development of ZhihuLive promising and offered good suggestions for the application procedure, such as increasing the in-depth field knowledge, providing better growth channels for content, expanding the breadth of the content, diversifying and simplifying payments, and so forth.

ZhihuLive caters to the features of the modern Internet era, including adopting fragmentation time and knowledge payments and possessing good application performance and development prospects. However, it also has some deficiencies that should be upgraded, and it can further improve its structure and user experience during the growth process.

We invited the participants to freely state their opinions on the factors needed to enhance knowledge payment behaviors. They consistently described and confirmed the key factors of self-efficacy, personal outcome expectations, community-related outcome expectations, social interaction relations, shared languages, shared visions, trust, and identification to promote participation in knowledge payment behaviors. The interview statements are described as follows:

Self-efficacy (SE) is a dynamic construct that reflects more than just an ability assessment. An individual’s judgment of SE reflects an orchestration or mobilization component that includes motivational and integrative aspects [24,41]. As one interviewee said, “I am able to gain knowledge in ZhihuLive, and the ability to easily use it is a matter of great concern to me.”

The notion that task completion leads to a certain consequence is referred to as outcome expectations. In this research, the community-related result expectation is ZhihuLive knowledge payers’ judgment of the possible implications of their knowledge-paying behavior on the virtual community. The personal result expectation refers to the judgment that the ZhihuLive knowledge payer may face consequences for their knowledge payment behavior. One interviewee stated, “I had made a lot of friends in the process of learning with ZhihuLive and can study happily. My participation can help the development of the community.”

[42] investigated social interactions (network ties) as information and resource flow routes. According to [32], relationship strength is a combination of time invested, intense emotions, closeness (mutual confidence), and reciprocal services. An interviewee remarked, “In the process of learning with ZhihuLive, I receive prompt communication from the knowledge provider to address my doubts. At the end of my studies, I will take the time to listen to the communication again.”

Within management literature, trust is viewed as a set of specific beliefs dealing primarily with the integrity, benevolence, and ability of another party [43]. The focus of this research is on integrity, referring to a person's expectation that individuals in a digital world will adhere to a set of widely accepted values, standards, and principles.

[16] define identification as "one's sense of self in terms of the defining elements of a personality social category," in the case of the virtual world. Identification is how individuals regard themselves as one with another person or group [11]. In this research, identification is a person’s sense of belonging and favorable feelings toward the digital world, comparable to emotional identification, as suggested by [44].

In the interview comments, one female participant said, “I paid money to sponsor this live, mainly because I wanted to get close to Big V and listen to the voice of Gejin. In the warm-up time a few days before the official start of the live event, Gejin was actively answering the questions of the live audience. It was almost a question and answer. No matter how many simple questions are answered seriously, it can be said that it is very conscientious. ”

Shared language addresses “the acronyms, subtleties, and underlying assumptions that are the staples of day-to-day interactions” [16]. A shared vision, according to [42], "embodies the collective ambitions and aspirations of an organization's members" and is "A bonding technique that allows diverse areas of a company to merge or combine resources" [15].

One interviewee stated, “I used to participate in a disappointing live broadcast; from the beginning to the end, it was merely a live broadcast of the host’s meal in which a group of people was talking without any logic, so it could only get a negative score.”

Another interviewee said, “Decoration is an area of high information asymmetry. The most valuable thing about this Live for me is that the speaker taught me the order of homework in the early stages of decoration and how to scientifically allocate time and energy.”

In line with the qualitative research, these opinions demonstrate the following: first, the participants provide user engagement factors from the perspective of the psychological level, including self-efficacy, personal consequence expectation, and community-orientation consequence expectation. This interesting phenomenon offers a critical point of entry for refining the final research model.

Next, with respect to the user experience and technology, the user experience and products involve all such aspects. Apart from offering professional content, it will reinforce technological research, development, and upgrading and provide a humanized service and experience to meet more users’ demands. In line with the interview results, the interviewees also expressed dissatisfaction with such matters as app design. For example, some interviewees proposed adding interactive user community elements, such as a messaging function, to facilitate discussion, which involves the horizontal and vertical structure of the app. At present, the pay-per-use content consists of simple published content or unidirectional instruction from tutors or experts, but vertical interactions between tutors and users or horizontal interactions among users are minimal, which causes a lack of community atmosphere, low attractiveness, and insufficient stickiness.

Finally, when referring to acceptable payment amounts, some users expressed that price would not be a concern if the learning content is what they need or is helpful. Others stated that with respect to affordability, they would readily accept a lower price, which is consistent with the research of [21].

Conceptual relationships related to the factors defined by qualitative research were incorporated into our research model, Figure 1. The model comprises the influencing factors of the individual psychological dimension and social capital, as well as the adjustment effect of price on knowledge payment. This was used to empirically test the relationship between these variables, as described in the next section.

- **"Introduction" and "Literature review" are clear, however the authors might think of giving a more precise definition of the theories they cite from time to time, rather than taking them for granted, for example "flow experience theory" (p. 3, line 91) and "theory of perceived value" (p. 5, line 179).**

Response: We appreciate the reviewer’s feedback, and we have added the suggested content to the manuscript on page 3, lines 109-110, and page 5, lines 191-192. Changes to the revised manuscript are highlighted in orange.

- **P. 6, lines 182-193, the authors state "Dou (2014) discovered that the utilised value of content items had a substantial impact on customers' payment behavior from the perspective of perceived risk". What do the authors mean by "perspective of perceived risk"?**

Response: Thank you for pointing this out. We clarify you regarding above meaning in the below:

Perceived risks refer to the spirit cost associated with customers' purchasing behavior, representing uncertainty about the future. This uncertainty will directly affect the consumers' purchase intention. Though we mentioned that the increase in knowledge payment allows users a new mode of knowledge sharing and communication, its impact on customers’ payment behavior is unclear.

We have clarified this point, as we will not modify this section in our manuscript.

- **P. 7, lines 245-249, the authors state "An individual's psychology has an influence on his or her willingness to share knowledge in the virtual community; in recent years, many studies have indicated that the self-efficacy index of knowledge sharing is the chief distinguishing feature of self-efficacy. Bock and Kim reveal that individuals' self-judgment of their contribution to an organization has a significant positive impact on knowledge sharing (Bock & Kim, 2002), while Atreyi, Tanbcy, and Wei also regard self-efficacy as an internal incentive to determine its effect on knowledge sharing (Atreyi et al., 2005)." It is not clear why the authors focus on the self-efficacy of knowledge providers and their knowledge sharing behavior if the focus of the research is on the sense of self-efficacy experienced by users (i.e., being confident of gaining relevant knowledge from the platform).**

Response: Thank you for this suggestion. It would have been interesting to explore this aspect. However, in our study, we focus on the self-efficacy of knowledge providers and their knowledge-sharing behaviors because more attention should be paid to enhancing a positive mood state for social associations. This precedes knowledge-sharing behaviors, and useful feedback should be provided to improve individual self-efficacy. When people share valuable expertise with an organization, they gain confidence in what they can do, increasing their self-efficacy. Knowledge self-efficacy is typically manifested in the form of people believing that their knowledge can help solve job-related problems, improve work efficiency, or make a difference in their organization.

- **P. 12, line 479, I think the authors should correct "variable inflation factor (VIF)" into "variance inflation factor (VIF)."**

Response: Thank you for pointing this out. We have corrected this to read "variance inflation factor (VIF)" in our revised manuscript, which can be found on page 16, line 631.

- **P. 13, "Hypotheses test results" paragraph. Did the authors control for the effect of demographic characteristics on the estimated paths in testing the structural relationships?**

Response: Thank you for this question. We have now clearly mentioned in our paper that the authors did not control for the effect of demographic characteristics on the estimated paths in the structural relationships.

- **P. 14, "Discussion and Conclusions" paragraph. Before discussing the theoretical and practical implications of the study, the results of the analyzes (including non-significant ones) should be discussed and justified. Instead, the authors just summarize them. The reader may be interested in why some relationships didn't work out, for example, the reasons why self-efficacy does not affect community-related outcome expectations and cognitive dimension. Therefore, I suggest arguing all the results obtained.**

Response: Thank you for this suggestion. We agree with the reviewer’s assessment. Accordingly, we have revised the “Discussion and conclusion” paragraph. The changed portion in the revised manuscript is highlighted in orange. For your consideration, we also copy and paste it here (this can also be found on pages 18-19, lines 706-757):

In this paper, the empirical results show a significant difference between self-efficacy & personal expectations and social capital structure. That is, in a Q&A community, the more confident a user is in their ability to complete a certain task or engage in a behavior, the more frequent communication they will have with others in the community, which will positively stimulate the individual’s expected results.

In the Q&A community, knowledge sharers should judge their abilities based on comprehensive evaluation criteria, including their ability to use the Q&A community and to express their opinions and expertise. The above analysis shows that self-efficacy is a form of self-assessment of the users' skills and confidence in completing particular behaviors. If the users are recognized for their shared information, they will be more confident and continue to share more knowledge.

The empirical analysis shows that community users’ self-efficacy significantly influences outcome expectations. Usually, those with higher self-efficacy will have more expectations of knowledge sharing. Research by Shang Yonghui, Ai Zhongqi, and Wang Fengyan on knowledge-sharing behaviors in virtual communities shows a significantly positive correlation between self-efficacy and outcome expectation.

In this paper, the empirical results show that outcome expectation significantly influences the cognitive level of social capital but does not significantly influence the.

The correlational research on the factors influencing knowledge sharing demonstrates that outcome expectation has no significant effect at the structural level. The two reasons for this are suggested to be that: (1) Q&A community members are weakly linked. As there is no face-to-face communication among members, it is difficult to establish good relationships, and it is easy for connections to be destroyed. The expected return on relationships, such as praise, support, respect, trust, internal satisfaction, or a sense of accomplishment from helping others, cannot be guaranteed; (2) within the community, users share knowledge without utilitarian purposes. Most knowledge-sharing behavior is done solely based on the value or characteristics of the knowledge itself.

In a knowledge-paying community based on common needs or interests, users share the same learning goals and values and form a consistent view of problems; thus, they generate a sense of identity through high-quality communications. The jargon and professional abbreviations used by content producers and users significantly affect building trust among users. In the knowledge-sharing behavior of virtual communities, the jargon and professional abbreviations can help users develop a more effective understanding of information.

Since specific professional sectors have their own unique professional terms, the application of these terms enables users to believe that the content is professional and thus builds their trust in the expertise of the content producer. On Zhihu, the knowledge community based on UGC (user-generated content), there are many professionals from different sectors. Since they generate professional content and common language used in discussions, professionals have a good reputation among users, leading to the users’ willingness to use Zhihu Live knowledge acquisition.

Shared language and vision have no significant effect on users' intention to pay. The possible reasons for this include: (1) The common language used by knowledge producers while generating content affects the users' perception of their professional competence, integrity, and goodwill. That is, trust will affect users' intent to pay for knowledge. (2) As an emerging sector, existing users pay for knowledge to solve problems and improve their capabilities. Therefore, among the factors affecting payment behavior, the users' judgment of the expertise of the knowledge producer is greater than the relationship between the users.

- **P. 16, "Limitations and future research" paragraph. Authors should recall the study's limitations, such as the use of self-report measures, the focus on intent to pay rather than actual user behavior, and the specificity of the target investigated (most participants were young adults and students), which could limit the generalizability of the results obtained.**

Response: Thank you. We think this is an excellent suggestion. We have already included the above suggestions in our revised manuscript, highlighted in orange. For your consideration, we have added three limitations here (which can be found on page 21, lines 828-850):

This paper provides theoretical support and corresponding business strategies for the development and operation of platforms by studying the factors affecting the willingness of online platform users. However, due to the limited scientific research capabilities and conditions, the study of related factors may be insufficient.

(1) Due to the constraints of time and space, this study used online questionnaires. Although relevant filling requirements were set to ensure the effectiveness of the questionnaire, virtuality and network uncertainty weakened the quality of the questionnaire, and the responder groups were somewhat limited. Therefore, future research should conduct questionnaires using more offline channels to ensure the objectivity and validity of the questionnaire data as much as possible.

(2) Although this study’s structural equation model is based on the results of previous scholars' research, and the data collected has been confirmed to be reliable and valid, the selection of model variables was made solely based on individual learning and research experience. Thus, the study has not yet determined all the variables that affect users' willingness to pay. Therefore, in future research and studies, research models on the influencing factors of users' willingness to pay should be further improved.

(3) The research on online knowledge service platforms is conducted from a macro perspective. In reality, online platforms have different operation methods. Therefore, this study lacks research on platform personality. Future relevant theoretical research can study a specific platform for more targeted exploration.

**Reviewer #2:**

**Major remarks**

- **It is not clear why Authors refer to mixed-method study if the entire manuscript presents only the results of a quantitative analysis. A couple of times it is cited a qualitative study, which outcomes however are never depicted. I invite Authors to provide full information about the qualitative study (and its contribution to the research development) or to modify the title (and narrative of the manuscript), clarifying the utility (and inputs) of the qualitative study.**

Response: Thank you for pointing this out. It would have been interesting to explore this aspect. We have conducted a mixed-method study. We have included our explanation regarding the qualitative method in the methodology section (highlighted in orange). We have copied and pasted the explanation here for your consideration (it can also be found on lines 393-538 on pages 10-14):

In this study, qualitative research was used to study the same problem using different methods so that the researchers could improve their self-confidence in the accuracy of the research results. Using qualitative research methods, a complete description of the research questions is provided to optimize the results.

Interviews are one of the most common qualitative data collection approaches, enabling researchers to gain deep insights from rich narratives [36]. Following [37], to promote data reliability in a qualitative study, the interviewer asked each question in a prescribed order. Meanwhile, to ensure inferential validity, including interpretive validity and confirmability, we attempted to accurately understand interviewees’ thoughts, views, feelings, and intentions by analyzing their discussions as recorded during the interview. We also confirmed and corroborated the interview results by cross-checking them with two colleagues to ensure their confirmability.

We used FGI to gather data on ZhihuLive users in China. We displayed posters and spread social recommendations to attract users to participate in our discussion. The focus group consisted of six users, which helped encourage a meaningful conversation [38]. The respondents consisted of four male and two female users who had 3 to 18 months of service experience, indicating that they are very familiar with the various situations in which the service is used. Their ages ranged from 20 to 30 years, representing the majority of service users. Also, the informants included university students, government and company employees, and an individual business owner.

We carefully designed the interview following [39]. The interview was carried out by a marketing lecturer and was conducted in a relaxed atmosphere in which participants were encouraged to talk openly.

The interview was divided into three sections. In the first part, we invited participants to share their service use experiences. The purpose of this initial stage was to arouse informants’ enthusiasm through a light-hearted question and determine how they thought and felt about the ZhihuLive service. In the second section, we discussed the particulars of the ZhihuLive platform and their perspectives on service through the lens of individual psychology. In the third section, we discussed the factors that influence the use of knowledge payment services, their frequency of use, and recommendations for any business model innovations. The insights from this section contribute to the subsequent development of hypotheses based on theoretical rationales from prior research.

The interview lasted nearly one hour and was smartphone-recorded and transcribed verbatim. Following the principles of [40], the content analyses of the recording and transcript were performed separately by two colleagues unaware of the specific research framework and hypotheses to avoid potential bias.

According to the interviews and research, ZhihuLive is characterized by applications in a short time, effective utilization of this time, and an ability to provide valuable information to users to solve their problems and assist them in learning new knowledge. The users found the development of ZhihuLive promising and offered good suggestions for the application procedure, such as increasing the in-depth field knowledge, providing better growth channels for content, expanding the breadth of the content, diversifying and simplifying payments, and so forth.

ZhihuLive caters to the features of the modern Internet era, including adopting fragmentation time and knowledge payments and possessing good application performance and development prospects. However, it also has some deficiencies that should be upgraded, and it can further improve its structure and user experience during the growth process.

We invited the participants to freely state their opinions on the factors needed to enhance knowledge payment behaviors. They consistently described and confirmed the key factors of self-efficacy, personal outcome expectations, community-related outcome expectations, social interaction relations, shared languages, shared visions, trust, and identification to promote participation in knowledge payment behaviors. The interview statements are described as follows:

Self-efficacy (SE) is a dynamic construct that reflects more than just an ability assessment. An individual’s judgment of SE reflects an orchestration or mobilization component that includes motivational and integrative aspects [24,41]. As one interviewee said, “I am able to gain knowledge in ZhihuLive, and the ability to easily use it is a matter of great concern to me.”

The notion that task completion leads to a certain consequence is referred to as outcome expectations. In this research, the community-related result expectation is ZhihuLive knowledge payers’ judgment of the possible implications of their knowledge-paying behavior on the virtual community. The personal result expectation refers to the judgment that the ZhihuLive knowledge payer may face consequences for their knowledge payment behavior. One interviewee stated, “I had made a lot of friends in the process of learning with ZhihuLive and can study happily. My participation can help the development of the community.”

[42] investigated social interactions (network ties) as information and resource flow routes. According to [32], relationship strength is a combination of time invested, intense emotions, closeness (mutual confidence), and reciprocal services. An interviewee remarked, “In the process of learning with ZhihuLive, I receive prompt communication from the knowledge provider to address my doubts. At the end of my studies, I will take the time to listen to the communication again.”

Within management literature, trust is viewed as a set of specific beliefs dealing primarily with the integrity, benevolence, and ability of another party [43]. The focus of this research is on integrity, referring to a person's expectation that individuals in a digital world will adhere to a set of widely accepted values, standards, and principles.

[16] define identification as "one's sense of self in terms of the defining elements of a personality social category," in the case of the virtual world. Identification is how individuals regard themselves as one with another person or group [11]. In this research, identification is a person’s sense of belonging and favorable feelings toward the digital world, comparable to emotional identification, as suggested by [44].

In the interview comments, one female participant said, “I paid money to sponsor this live, mainly because I wanted to get close to Big V and listen to the voice of Gejin. In the warm-up time a few days before the official start of the live event, Gejin was actively answering the questions of the live audience. It was almost a question and answer. No matter how many simple questions are answered seriously, it can be said that it is very conscientious. ”

Shared language addresses “the acronyms, subtleties, and underlying assumptions that are the staples of day-to-day interactions” [16]. A shared vision, according to [42], "embodies the collective ambitions and aspirations of an organization's members" and is "A bonding technique that allows diverse areas of a company to merge or combine resources" [15].

One interviewee stated, “I used to participate in a disappointing live broadcast; from the beginning to the end, it was merely a live broadcast of the host’s meal in which a group of people was talking without any logic, so it could only get a negative score.”

Another interviewee said, “Decoration is an area of high information asymmetry. The most valuable thing about this Live for me is that the speaker taught me the order of homework in the early stages of decoration and how to scientifically allocate time and energy.”

In line with the qualitative research, these opinions demonstrate the following: first, the participants provide user engagement factors from the perspective of the psychological level, including self-efficacy, personal consequence expectation, and community-orientation consequence expectation. This interesting phenomenon offers a critical point of entry for refining the final research model.

Next, with respect to the user experience and technology, the user experience and products involve all such aspects. Apart from offering professional content, it will reinforce technological research, development, and upgrading and provide a humanized service and experience to meet more users’ demands. In line with the interview results, the interviewees also expressed dissatisfaction with such matters as app design. For example, some interviewees proposed adding interactive user community elements, such as a messaging function, to facilitate discussion, which involves the horizontal and vertical structure of the app. At present, the pay-per-use content consists of simple published content or unidirectional instruction from tutors or experts, but vertical interactions between tutors and users or horizontal interactions among users are minimal, which causes a lack of community atmosphere, low attractiveness, and insufficient stickiness.

Finally, when referring to acceptable payment amounts, some users expressed that price would not be a concern if the learning content is what they need or is helpful. Others stated that with respect to affordability, they would readily accept a lower price, which is consistent with the research of [21].

Conceptual relationships related to the factors defined by qualitative research were incorporated into our research model, Figure 1. The model comprises the influencing factors of the individual psychological dimension and social capital, as well as the adjustment effect of price on knowledge payment. This was used to empirically test the relationship between these variables, as described in the next section.

- **Clarify the sample characteristics. It is currently reported as “non-random”, does it mean it is a convenience sample? Please state so. Additionally, in the results section (sample profile) it is stated [lines 456-457]: “The majority of the students in the sample (n = 349) studied for a Bachelor's degree or college.” Which leads readers to think that the sample is entirely formed by students (partially supported by the overwhelming majority of respondents aged between 20 and 39). Is this the case?** **Furthermore, the shortcomings stemming from a non-representative sample should be clearly reported in the results, discussion and limitations sections.**

Response: Thank you for your comment. We have clearly mentioned in our manuscript that to avoid a homogeneous sample population, the author distributed it to WeChat users from differing relationships, industries, education levels, and ages through snowball diffusion on the WeChat platform.

While we appreciate the reviewer’s feedback, we respectfully disagree that our sample is not partially supported by the overwhelming majority of the respondents because students do not entirely form our sample. We explained how we collected our data in our manuscript. To avoid a homogeneous sample population, the author distributed it to WeChat users from differing relationships, industries, education levels, and ages through snowball diffusion on the WeChat platform. A total of 538 questionnaires were collected in this survey. Furthermore, the shortcomings stemming from a non-representative sample have been clearly reported in the results, discussion, and limitations sections.

**Minor remarks**

- **I suggest that the manuscript receives professional English editing (several sentences are quite unclear, and the development is not easy to grasp).**

Response: Thank you for this suggestion and for pointing this out. An English editing service has revised the article. The corrections can be found in the revised manuscript using the track changes function.

- **Having seventeen research hypotheses, I would strongly advice Authors to add a summary table reporting the acceptance or rejection of each hypothesis.**

Response: Thank you for this suggestion. We have added a summary table reporting the acceptance or rejection of each hypothesis to the revised manuscript, highlighted in orange. For your consideration, please see Table 6 below (it can also be found on lines 683-685 and 1185-1187 on pages 17 and 38):

Table 6. Summary Table Reporting the Acceptance or Rejection of Each Hypothesis

| **Hypothesis** | **Path** | **Beta Score** | **Result** |
| --- | --- | --- | --- |
| **H1** | SE->POE | β= 0.107, p<0.05 | Accepted |
| **H2** | SE->COE | β= 0.032, p>0.05 | Rejected |
| **H3** | SE->SL | β= -0.029, p>0.05 | Rejected |
| **H4** | SE->SV | β= 0.053, p>0.05 | Rejected |
| **H5** | SE->SI | β= 0.134, p<0.001 | Accepted |
| **H6** | POE->SL | β= 0.549, p<0.001 | Accepted |
| **H7** | POE->SV | β= 0.064, p<0.05 | Accepted |
| **H8** | POE->SI | β= 0.089, p<0.05 | Accepted |
| **H9** | COE->SL | β= 0.256, p<0.001 | Accepted |
| **H10** | COE->SV | β= 0.532, p<0.001 | Accepted |
| **H11** | COE->SI | β= 0.061, p>0.05 | Rejected |
| **H12** | SL->KP | β= 0.273, p<0.001 | Accepted |
| **H13** | SL->TR | β= 0.092, p<0.05 | Accepted |
| **H14** | SL->ID | β= 0.255, p<0.001 | Accepted |
| **H15** | SV->TR | β= 0.200, p<0.001 | Accepted |
| **H16** | SV->KP | β= -0.188, p<0.001 | Accepted |
| **H17** | SV->ID | β= 0.274, p<0.001 | Accepted |
| **H18** | SI->TR | β= 0.157, p<0.001 | Accepted |
| **H19** | SI->ID | β= 0.230, p<0.001 | Accepted |
| **H20** | SI->KP | β= 0.278, p<0.001 | Accepted |
| **H21** | TR->KP | β= 0.204, p<0.001 | Accepted |
| **H22** | ID->KP | β= 0.045, p>0.05 | Rejected |
| **H23** | PR->KP | β= 0.018, p>0.05 | Rejected |
| **H24** | PR x SL->KP | β= -0.522, p<0.001 | Accepted |
| **H25** | PR x TR->KP | β= 0.195, p<0.001 | Accepted |
| **H26** | PR x ID->KP | β= -0.086, p<0.05 | Accepted |
| **H27** | PR x SI->KP | β= 0.086, p>0.05 | Rejected |

- **The authors should provide a thorough description of the core limitations of the research (in the current version, only future research is discussed).**

Response: Thank you. We think this is an excellent suggestion and have included this in our revised manuscript, highlighted in orange. For your consideration, we have added three limitations here (which can also be found on page 21, lines 828-850):

This paper provides theoretical support and corresponding business strategies for the development and operation of platforms by studying the factors affecting the willingness of online platform users. However, due to the limited scientific research capabilities and conditions, the study of related factors may be insufficient.

(1) Due to the constraints of time and space, this study used online questionnaires. Although relevant filling requirements were set to ensure the effectiveness of the questionnaire, virtuality and network uncertainty weakened the quality of the questionnaire, and the responder groups were somewhat limited. Therefore, future research should conduct questionnaires using more offline channels to ensure the objectivity and validity of the questionnaire data as much as possible.

(2) Although this study’s structural equation model is based on the results of previous scholars' research, and the data collected has been confirmed to be reliable and valid, the selection of model variables was made solely based on individual learning and research experience. Thus, the study has not yet determined all the variables that affect users' willingness to pay. Therefore, in future research and studies, research models on the influencing factors of users' willingness to pay should be further improved.

(3) The research on online knowledge service platforms is conducted from a macro perspective. In reality, online platforms have different operation methods. Therefore, this study lacks research on platform personality. Future relevant theoretical research can study a specific platform for more targeted exploration.

- **No information on ethical approval (or waiver) of the study is currently provided in the manuscript, neither information on the collection of informed consent from respondents (or any other ethical guideline followed by the researchers).**

Response: Institutional Review Board approval is required. However, we indicated that ethical approval is not necessary for our study because we strongly believe that ethical review is only needed if researchers are unable to obtain consent, if the case report contains sensitive information, or if there is a potential risk to patient privacy. We do not consider such sensitive information in our research. We strongly believe that the use of existing data collections or records that contain only non-identifiable data, e.g., survey response data, publicly accessible records, archives, or publications, are exempted from ethical approval. Anyway, we have already got ethical approval from our institution that we include for your kind consideration. The number is as follow:

1040198-230207-HR-010-02

6. **PLOS authors have the option to publish the peer review history of their article (what does this mean?<****https://journals.plos.org/plosone/s/editorial-and-peer-review-process#loc-peer-review-history****>). If published, this will include your full peer review and any attached files.
   If you choose “no”, your identity will remain anonymous but your review may still be made public.
   Do you want your identity to be public for this peer review? For information about this choice, including consent withdrawal, please see our Privacy Policy<https://www.plos.org/privacy-policy>.
   Reviewer #1: No
   Reviewer #2: No**

Response: Thank you for pointing this out. We agree to choose “Yes” so that we have the option to publish the peer review history in our article.
